# A Continuum Damage Mechanics Model for the Static and Cyclic Fatigue of Cellular Composites

**DOI:** 10.3390/ma10080951

**Published:** 2017-08-15

**Authors:** Sergej Diel, Otto Huber

**Affiliations:** 1 Audi AG, D-85045 Ingolstadt, Germany; Sergej.Diel@gmx.de; 2 Competence Center for Lightweight Design (LLK), University of Applied Sciences Landshut, D-84036 Landshut, Germany

**Keywords:** cellular composite, syntactic foam, fatigue damage modeling, continuum damage mechanics, frequency effect, mean stress dependency

## Abstract

The fatigue behavior of a cellular composite with an epoxy matrix and glass foam granules is analyzed and modeled by means of continuum damage mechanics. The investigated cellular composite is a particular type of composite foam, and is very similar to syntactic foams. In contrast to conventional syntactic foams constituted by hollow spherical particles (balloons), cellular glass, mineral, or metal place holders are combined with the matrix material (metal or polymer) in the case of cellular composites. A microstructural investigation of the damage behavior is performed using scanning electron microscopy. For the modeling of the fatigue behavior, the damage is separated into pure static and pure cyclic damage and described in terms of the stiffness loss of the material using damage models for cyclic and creep damage. Both models incorporate nonlinear accumulation and interaction of damage. A cycle jumping procedure is developed, which allows for a fast and accurate calculation of the damage evolution for constant load frequencies. The damage model is applied to examine the mean stress effect for cyclic fatigue and to investigate the frequency effect and the influence of the signal form in the case of static and cyclic damage interaction. The calculated lifetimes are in very good agreement with experimental results.

## 1. Introduction

Cellular composites are composite foams with cellular granules as place holders in a polymer or metal matrix material. The place holders consist of glass, mineral, or metal closed-cell foams [1,2]. When using appropriate material combinations for the matrix and place holders, the cellular place holders improve the weight-specific mechanical properties of the cellular composite. The investigated cellular composite consists of cellular glass granules from recycled glass embedded in an epoxy matrix. The material offers high weight-specific stiffness and compressive strength at low material costs [1,3,4]. Cellular composites are manufactured by the infiltration of granules with the matrix material via a casting process [5].

When using cellular composites in structural components, knowledge of the mechanical properties and numerical modeling of the material is necessary. The elastic, visco-elastic, and elastic-plastic behavior of cellular composites is analyzed and modeled in [1,4,6,7]. Klaus et al. [1] developed rheological models to describe the effective macroscopic material properties stiffness and strength for uniaxial loading by the properties of the basic materials. These models are modified Voigt–Reuss mixing rules, in which the influence of the interface between the matrix and the granule is taken into account. The extension of the model by viscous properties of the matrix material enables the simulation of the visco-elastic behavior of cellular composites [4]. In combination with an idealized finite element mesomodel for cellular composites, the parameters of the rheological model are determined by the properties of the single material’s components. Therefore, material properties such as stiffness and the uniaxial static strength of a cellular composite may be determined without material tests on the cellular composite. The yield criterion for a three-dimensional (3D) stress state was experimentally determined by uniaxial tensile and compressive tests, shear tests, and hydrostatic compression tests and modeled using the elliptic yield condition by Nusholtz et al. [8] in [3,6]. Bartl [7] analyzed a cellular composite with a PA6 matrix by uniaxial tests at finite strains and high strain rates, and developed an elastic-plastic constitutive law on the basis of [8]. This material model was implemented into the finite element method (FEM) code LS-DYNA and validated by several crash applications.

The static and fatigue behavior of the presented cellular composite (recycled glass foam granules in epoxy resin) is analyzed in [9,10,11] for uniaxial compression and tension. Epoxy resin enables a good connection with glass granules without specific coatings. The quasi-static shear properties of this cellular composite are investigated in [11] using a new shear test fixture, which is particularly suitable for the testing of cellular solids [12]. This shear test fixture is an extended version of a picture-frame shear fixture, and allows for comparatively thick rectangular block specimens. In the small-strain regime, the fixture deforms nearly consistently with the definition of pure shear [12]. Analytical and numerical homogenization methods for cellular composites with an ideal connection between granules and matrix are shown in [9,13]. Using these methods, the effective linear elastic properties can be calculated in a very effective manner from the elastic constants of the constituent materials without tests on the composite.

Three examples of practical applications demonstrate the high lightweight capability of cellular composites. The lightweight potential of thin-walled, closed-hat members, partially supported by hollow cores consisting of glass foam granules and a two-component epoxy resin, is investigated in [14]. Nonlinear finite element analyses are applied to simulate the behavior under quasi-static three-point bending and to estimate the lightweight potential. The simulations are validated by experiments on thin-walled side members of the chassis of a recreational vehicle with and without supporting cores in [14]. The results show a significant potential for weight reduction by using cellular supporting cores. Experiments have shown that the weight-specific collapse load is 38% larger in comparison to thin-walled members without supporting cores. In [6,7] a cellular composite was used as a supporting core in a thin-walled steel deformation element of the front area of a car. The hybrid deformation element results in higher energy absorption and a smoother force-displacement curve compared to monolithic steel deformation elements with a higher wall thickness.

The application of a cellular composite in a sandwich structure is shown in [15]. Using cellular composites as core material, it is possible to produce sandwich structures with fiber-reinforced composites as face layers in one step [16]. The idea is to eliminate the adhesive lamination process between a core and the surface layers by integrating the surface-layer production into the manufacturing process of the core. To demonstrate the advantages of the proposed cast process, a 3D-shaped sandwich skateboard with a cellular composite core has been developed [11]. A cellular composite with 0.5–1 mm foam granules made from recycled glass was chosen as the core material, and carbon fiber reinforced layers are chosen as the face material. The matrix material is epoxy resin [11,15]. Due to the complex structure of the skateboard, the manufacturing process is carried out with the help of an infiltration core as proposed in [5]. The weight of the sandwich skateboard could be reduced by 14% compared to a conventional skateboard made of plywood. While maintaining similar stiffness, the static strength of the sandwich board could be increased by 79% [15].

Since sandwich structures like skateboards or thin-walled hybrid members for car structures are loaded by different load-time functions, the static and cyclic damage behavior of the cellular composite has to be analyzed. Only research works for the fatigue behavior of conventional syntactic foams with hollow microspheres are known from the published literature. Ferreira et al. [17] analyzed the influence of a short fiber reinforced syntactic foam with an epoxy resin matrix under cyclic three-point bending. The fatigue behavior of a fire resistant syntactic foam with a phenol-formaldehyde resole resin matrix and cenospheres as place holders is investigated by Hossain and Shivakumar by cyclic compression, shear, and bending tests [18,19,20]. Further investigations on the fatigue behavior of syntactic foams are not known.

In this paper, the static and the cyclic damage behavior of the cellular composite (recycled glass foam granules in an epoxy resin matrix) is analyzed and modeled for different amplitudes, mean stresses, waveforms, and frequencies. As a basis for the lifetime prediction of the core material, a continuum damage mechanics model for uniaxial static and cyclic loading is developed. The damage is separated into pure static and pure fatigue damage, and is described in terms of the stiffness loss of the material. The damage evolution is modeled as a superposition of static and cyclic damage. The model incorporates the nonlinear accumulation and interaction of damage. A comparison with experimental results is given.

Since the analyzed cellular composites consist of the same base materials (epoxy resin and glass) with a similar outer shape of the place holders (cellular grains instead of hollow spheres), the proposed damage models might be suitable for conventional syntactic foams with hollow glass spheres as well.

## 2. Mechanical Properties and Fatigue Behavior

### 2.1. Constituent Materials

The cellular composite described in [9,10,11] was manufactured from epoxy laminating resin EPIKOTE™ MGS LR 285 (Hexion Speciality Chemicals GmbH, Iserlohn-Letmathe, Germany) and EPIKURE™ MGS 286 curing agent (Hexion Speciality Chemicals GmbH, Iserlohn-Letmathe, Germany) as matrix material by the low pressure infiltration (0.03–0.05 MPa) of a mold filled with granules. Glass foam granules with the grain-size fraction 1–2 mm (Dennert Poraver GmbH, Schlüsselfeld, Germany) are used as filler material. No coupling agent is used. The mechanical properties of the constituent materials can be found in [9].

**Figure 1:**
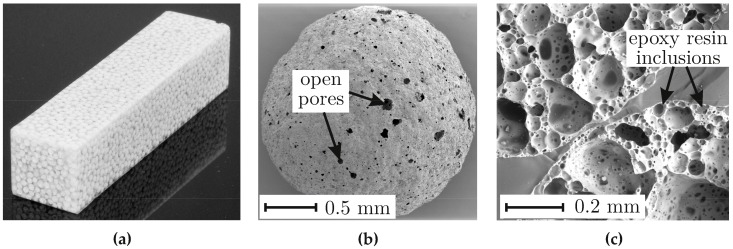
(**a**) Sample of a cellular composite with 1–2 mm glass foam granules as place holders and epoxy resin matrix; (**b**) SEM image of a glass foam grain; (**c**) SEM image of the fracture surface of a cellular composite with 1–2 mm glass foam granules, taken from a tensile test (from [9] by permission of Springer Science + Business Media).

Figure 1a shows a sample of a cellular composite with 1–2 mm glass foam granules and an epoxy resin matrix. The outer surface of a glass foam grain produced from recycled soda-lime glass is shown in Figure 1b. There are isolated open pores on the surface of the grain, which result from the manufacturing process. During the mold process, the epoxy resin can penetrate into the grains through these pores (see Figure 1c) and create an additional local form-fit with the grain.

### 2.2. Quasi-Static Mechanical Properties

The quasi-static mechanical properties of cellular composites with different sizes of glass foam granules were discussed in [13,15]. Table 1 contains the mechanical properties of the cellular composite with 1–2 mm glass foam granules as used in the following investigation. The yield strength σ_y_ is defined as stress at 0.1% plastic strain.

The failure behavior under quasi-static loading is brittle, with failure occurring perpendicular to the loading axis in tension, with failure at about 1% strain. In compression, the final failure occurs due to strain localization along a shear band at approximately 45° to the loading axis by failure strains of about 20%. As is typical for brittle materials, the strength in tension is considerably lower than in compression, see Table 1.

### 2.3. Static and Cyclic Fatigue Behavior

The fatigue behavior of composites depends on the mechanical properties of the constituent materials, the interface, and the interactions between the constituents. Epoxy resin exhibits time-dependent behavior due to visco-elasticity. When subjected to static loading, it deforms continuously and creep rupture may occur [21,22]. Under cyclic loading, epoxy resin may also fail due to crack initiation and propagation [23]. An associated cyclic creep deformation may be present in cyclic tests at mean stresses σ_m_ ≠ 0 [24].

The lifetime of glass depends mainly on the time under load. This effect is called static fatigue or delayed fracture [25,26], and is caused by stress corrosion under the influence of water vapor and applied load. Glass foams are also sensitive to the phenomenon of static fatigue [27,28]. Additionally, it was shown in [29] that cyclic fatigue can also occur in glass to a smaller extent compared to static fatigue. To the knowledge of the authors, there have not been published any studies about the cyclic fatigue of glass foams yet.

**Figure 2:**
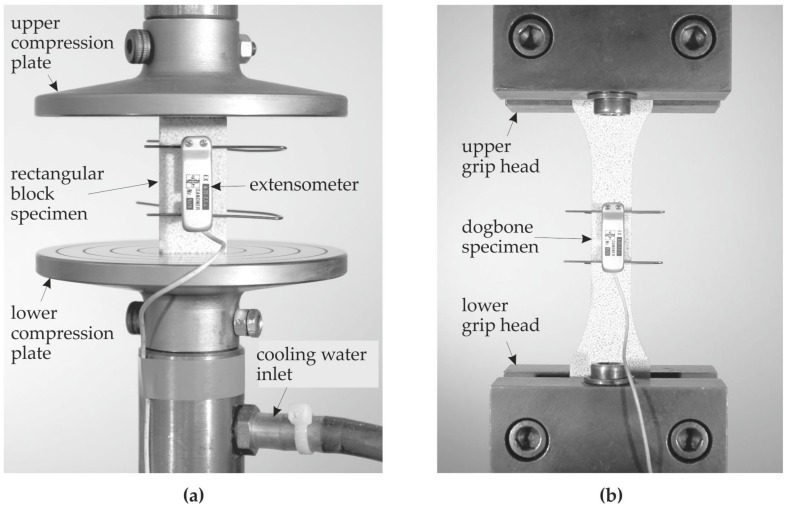
Test setup for (**a**) compressive test, and (**b**) tensile and reversal tests.

The fatigue behavior of cellular composites under uniaxial static and cyclic tensile, compressive, and reversal loading was investigated in [9,10,13]. Figure 2 shows the test setup. Further details concerning the experiments and their evaluation can be found in [13].

The static damage behavior was analyzed for stress ranges 0.4 < σ_max_/σ_y,T_ ≤ 1.4 in tension and 0.66 < |σ|_max_/σ_y,C_ ≤ 0.91 in compression. For the cyclic damage, the stress ranges 0.4 < σ_max_/σ_y,T_ ≤ 0.91 in tension and 0.66 < |σ|_max_/σ_y,C_ ≤ 0.91 in compression were used. The stress ratios R and the frequencies were also varied. It was found that the damage process under cyclic loading is an interaction between the static and the cyclic damage.

**Figure 3:**
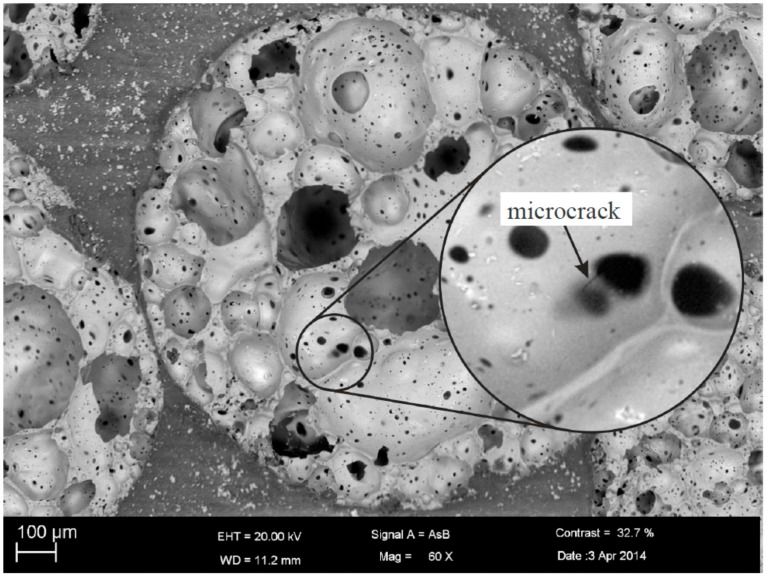
Microcrack in the glass foam grain of a virgin cellular composite specimen.

The damage behavior was analyzed by means of scanning electron microscopy (SEM, Carl Zeiss Microscopy GmbH, Jena, Germany). It was found that there are individual microcracks in the glass foam grains even in virgin specimens (see Figure 3). Investigations have further shown that microcracks grow in the glass foam granules first, when the cellular composite is subjected to mechanical loading.

**Figure 4:**
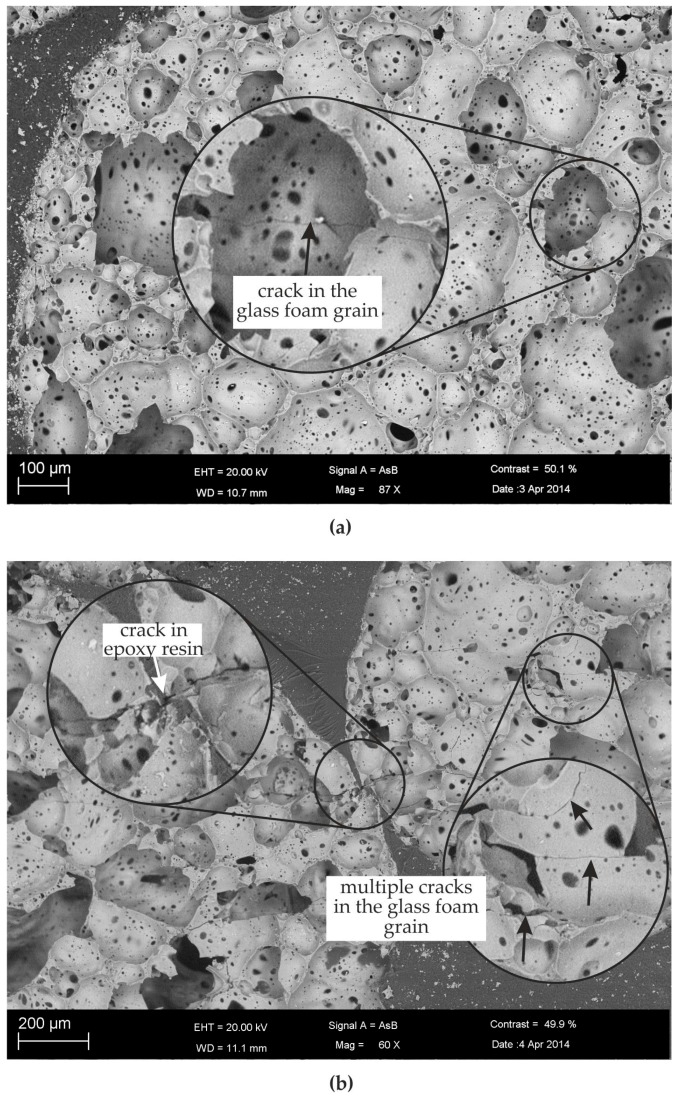
SEM image of the lateral surface of a cyclic damaged specimen after fracture (loading in vertical direction) for (**a**) cyclic tensile loading at σ_max_/σ_y,T_ = 0.5 and R = 0.1, and (**b**) static tensile loading at σ_max_/σ_y,T_ = 0.82.

Microcracks as shown in Figure 3 are supposed to be starting points for further crack growth through the glass foam grains. Subsequently, the microcracks lead to the formation of a macrocrack passing through the glass foam grains and the matrix. The failure of the specimen is finally caused by these macrocracks. In case of tensile loading, the final crack density depends on the applied load level. Figure 4a shows the crack distribution in a glass foam grain of a specimen subjected to a low cyclic tensile loading by the load level σ_max_/σ_y,T_ = 0.5 and R = 0.1. A crack passing through the grain can be seen. At low load levels, only a few cracks were found in single grains of the tested specimens. The cracks are oriented primarily perpendicular to the loading direction.

At higher loads, the crack density becomes higher. Figure 4b shows the microstructure of a specimen tested in a static tensile test at σ_max_/σ_y,T_ = 0.82. It was found that, in this case, almost all of the grains have several cracks. Small cracks were also observed in the matrix walls between two adjacent grains.

**Figure 5:**
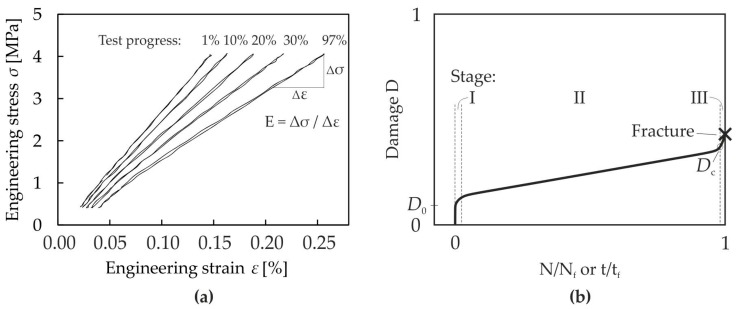
(**a**) Typical stress-strain hysteresis loops obtained from a cyclic tensile test (R = 0.1) at 20 Hz and σ_max_/σ_y,T_ = 0.7; (**b**) Schematic of a typical damage development during static and cyclic tests.

Typical stress-strain hysteresis loops obtained from a cyclic tensile test at a 20 Hz test frequency and σ_max_/σ_y,T_ = 0.7 are shown in Figure 5a. In general, a permanent decrease in stiffness was observed with ongoing damage in all specimens.

As a result of the crack growth, there is a reduction of stiffness in the specimen. The damage D is therefore described in terms of macroscopic stiffness loss (see e.g., [30])

(1) $$D=1-\frac{E}{E_{0}},$$

where E is the current Young’s modulus. E_0_ is the initial Young’s modulus of the undamaged specimen, which is defined as the initial stiffness of the first unloading path of the initial σ-ε hysteresis loop at low stresses. Figure 5b shows a schematic of a typical damage evolution curve. This kind of damage evolution behavior is typical for static and for cyclic loading. In the first stage, the damage rate is high. An initial damage D_0_ may occur immediately depending on the applied load level. During Stage II, which is the longest stage, the damage rate is low. In the final Stage III, the damage rate increases again and a macrocrack develops in the specimen, leading to the final failure of the specimen. The critical value of damage prior to fracture is called D_c_ (see e.g., [30]).

**Figure 6:**
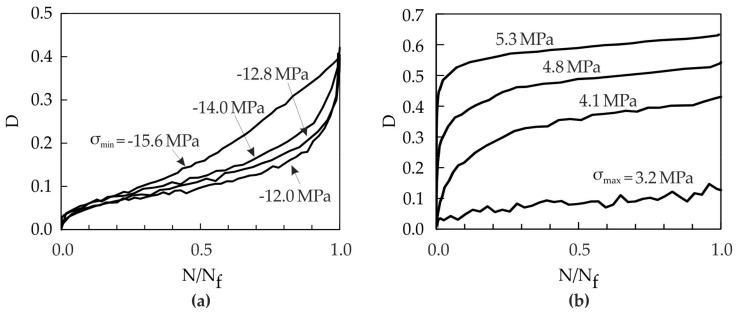
Experimental results of typical damage evolution curves for pure cyclic damage: (**a**) Compressive loading (R = 10, 30 Hz, triangular waveform); (**b**) Tensile loading (R = 0.1, 20 Hz, sinusoidal waveform).

The damage evolution behavior is different for tension and compression loading [10]. In tensile tests, the main damage growth occurs in Stages I and II. The damage evolution in Stage III is very low. In contrast, the main damage in compressive tests develops in Stages II and III (see Figure 6). This different damage behavior will be taken into account within the damage modeling in Section 3.

**Figure 7:**
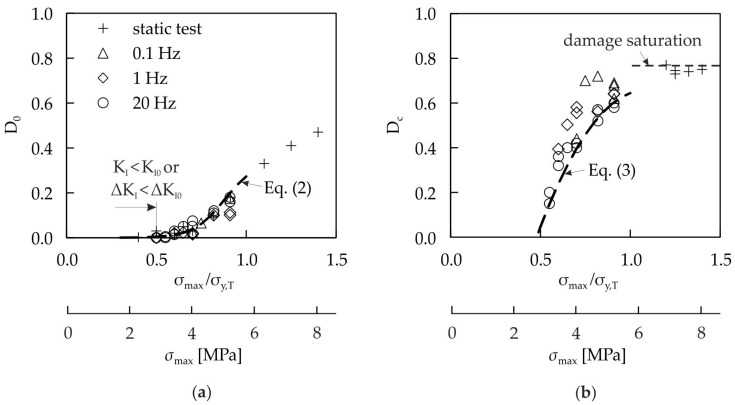
Values of initial damage D_0_ (**a**) and critical damage D_c_ (**b**) during static and cyclic tensile tests (R = 0.1) for different load levels and frequencies.

The damage evolution behavior differs not only for tension and compression, but is also dependent on the values of D_0_ and D_c_. In case of compression, D_0_ and D_c_ are nearly independent of the load level and can be set as constant to 0.05 and 0.4, respectively (see Figure 6a). When D_c_ is higher than 0.4, the material is considered to be completely damaged and not suitable for load carrying applications anymore. Such behavior in compression is different to that in tension as shown in Figure 6. Figure 7 shows the values of D_0_ and D_c_ as function of the applied load level determined during static tensile tests and cyclic tensile tests at different frequencies.

There is a good correlation of D_0_ as well as of D_c_ when they are described as a function of maximum stress for the static and cyclic tests. When the stress is sufficiently low, no initial damage develops. This behavior can be explained by the existence of a threshold stress intensity factor K_I0_ in static tests and ΔK_I0_ in cyclic tests. The threshold values K_I0_ and ΔK_I0_ depend on the crack length and density in the glass foam grains. These cracks are formed during cooling phases in the manufacturing process. When the applied stress intensity factor is lower than the threshold value, no cracks will develop in the glass foam. The critical damage D_c_ also depends on the load level, and can reach a maximum value of about 0.75 (see Figure 7b). This is a stage when all grains are damaged and the load is carried only by the matrix.

Different functions have been applied in order to approximate the initial damage and the critical damage. A tanh-function suits well for the description of the initial damage D_0_. In the range 0.0 < σ_max_/σ_y,T_ ≤ 1.0, D_0_ can be described by

(2) $$D_{0}=\tanh\left(\frac{\sigma_{\max}}{\mathrm{MPa}}-5.1\right)\;0.17+0.17.$$

Using Equation (2), the initial damage D_0_ is not exactly zero below σ_max_/σ_y,T_ = 0.5. However, the calculated damage is very low for these load levels so that this has no significant effect on the results of the damage model.

The critical damage D_c_ is approximated by the quadratic polynomial

(3) $$D_{c}=1-\left(0.054\;\;{\left(\frac{\sigma_{\max}}{\mathrm{MPa}}\right)}^{2}-\;\;0.676\;\;\left(\frac{\sigma_{\max}}{\mathrm{MPa}}\right)+2.46\right)$$

in the stress range 0.5 < σ_max_/σ_y,T_ ≤ 1.0. The specific numbers in Equations (2) and (3) result from a fitting process. For the derivation of Equation (3), only results at a 20 Hz test frequency are considered (see Figure 6b), which leads to a conservative fatigue life estimation. At load levels σ_max_/σ_y,T_ ≤ 0.5, no initial damage develops, and there was also no fracture observed. This can be explained by the threshold stress intensity factor, which is higher than the applied stress intensity factor in the specimen, which prohibits crack growth.

## 3. Damage Modeling and Parameter Identification

### 3.1. Static Damage Model

In this section, the damage evolution for static tensile and compressive uniaxial loading is modeled, and the parameter identification is carried out for a cellular composite with an epoxy resin matrix and 1–2 mm glass foam granules at room temperature. The load range is 0 ≤ σ_max_/σ_y,T_ ≤ 0.91 for tensile damage and 0 ≤ |σ|_max_/σ_y,C_ ≤ 0.91 for compressive damage. The parameters for the damage model are identified on the basis of the experimentally determined damage evolution in pure static damage tests.

The static damage model is based on creep and stress corrosion effects, which are time-dependent. The main influence quantities are the stress level and the amount of the current damage D. Kachanov [31] developed a damage model to calculate the time to creep rupture, when creep damage is accumulated in metals under uniaxial tension at higher temperatures. Rabotnov [32] extended the Kachanov model by the dependency of the damage rate on the current damage D. Using the Kachanov–Rabotnov model, the damage increment can be calculated by

(4) $$\mathrm{dD}=A\frac{\sigma^{m}}{{\left(1-D\right)}^{p}}\mathrm{dt}.$$

The parameter A, the stress exponent m, and the damage exponent p are material parameters. In this model, the damage increment depends on the current damage D. Hence, nonlinear damage evolution behavior is considered in the model.

For a constant stress σ together with the initial damage D(t = 0) = D_0_ and the fracture condition D(t = t_f_) = D_c_, the time to fracture t_f_ can be calculated by the integration of Equation (4) and results in

(5) $$t_{f}=\frac{{\left(1-D_{c}\right)}^{1+p}-{\left(1-D_{0}\right)}^{1+p}}{-A(1+p)\;\sigma^{m}}.$$

The current damage may be calculated at any time t and with the help of Equation (5), expressed by

(6) $$D=1-{\left({\left(1-D_{0}\right)}^{1+p}+\frac{t}{t_{f}}\left({\left(1-D_{c}\right)}^{1+p}-{\left(1-D_{0}\right)}^{1+p}\right)\right)}^{\frac{1}{1+p}}.$$

The damage evolution Equation (6) as a function of the relative lifetime t/t_f_ depends on the initial damage D_0_, the critical damage D_c_, and the damage exponent p. The identification of D_0_ and D_c_ is explained in Section 2.3. The identification of the damage exponent p is carried out in Section 3.1.1.

#### 3.1.1. Damage Exponent, p

**Figure 8:**
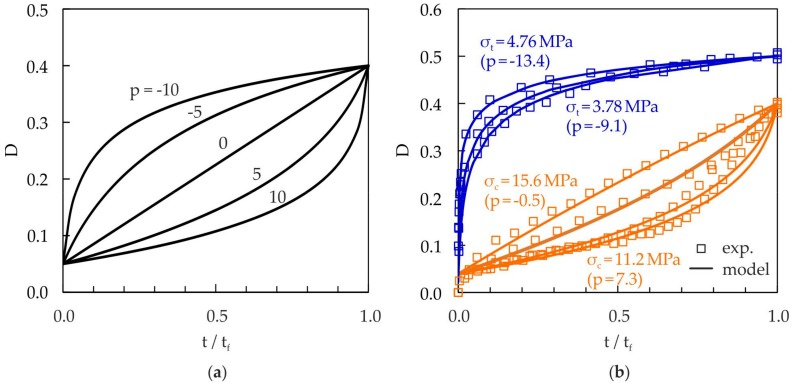
Influence of damage exponent p on the damage evolution curves: (**a**) damage development for different values of p, calculated by Equation (6) (D_0_ = 0.05, D_c_ = 0.4); (**b**) comparison of damage evolution curves for tensile and compressive loading (experiment and damage model).

The influence of the damage exponent p on the damage evolution is shown in Figure 8a. In order to describe nonlinear damage accumulation, p has to be a function of the stress p = p(σ), which was introduced by Lemaitre and Chaboche [33]. In the case of the investigated cellular composite, the damage evolution in terms of relative lifetime t/t_f_ depends on the applied stress level (see Figure 8b). This means that there is nonlinear damage accumulation, which leads to sequence effects. Negative damage exponents describe disproportionately high damage growth at the beginning of the lifetime, whereas positive damage exponents describe highly progressive damage growth at the end of the lifetime.

The damage exponent p is identified by static tensile and compressive tests at different stress levels, and is a linear function of the applied stress in the tensile range

(7) $$p=-4.5\;\frac{\sigma }{MPa}+8.0$$

as well as in the compressive range

(8) $$p=-1.8\;\frac{\left|\sigma \right|}{\mathrm{MPa}}+27.5.$$

Since specimen fracture could not be achieved in all static tensile experiments within an acceptable period of time, a critical damage D_c_ = 0.5 is chosen alternatively in order to identify the damage exponent p. This critical damage is reached for all tested stress levels, and the damage gradient becomes very small then (see Figure 8b). In the case of compressive loading, a critical damage D_c_ = 0.4 is chosen because the damage rate becomes very high when this value is reached, which leads to the very fast failure of the specimens.

In Figure 8b, the comparison of the modeled damage evolution with the experimental data is plotted for tensile and compressive static loading. A good agreement can be observed. The damage exponent p remains positive nearly over the complete compressive stress range. At higher compressive stresses, the damage exponent p becomes negative, which leads to a change in the curvature in the damage evolution curve (see Figure 8b). Almost over the entire tensile range, the damage exponent p is negative, whereby the most damage growth is at the beginning of the lifetime. Only at very low tensile stresses does the damage exponent become positive, and the damage evolution curve approaches the damage evolution of compressive loading.

#### 3.1.2. Stress Exponent, m

The slope of the creep strength line is determined by the stress exponent m. To identify the stress exponent m, a known procedure from cyclic damage is used. For cyclic fatigue damage, Chaboche and Lesne [34] described the damage increment dD as function of the current damage D, the stress amplitude σ_a_, the stress exponent β, the damage exponent α, and the mean stress parameter M, by

(9) $$\mathrm{dD}=D^{\alpha }{\left(\frac{\sigma_{a}}{M}\right)}^{\beta }\mathrm{dN}.$$

Chaboche and Lesne [34] proposed the evaluation of the cyclic stress exponent β in Equation (9) out of the experimentally determined Wöhler curve. The finite life fatigue strength can be described by the exponential function

(10) $$N_{f}=C\;\sigma_{a}^{-\gamma }$$

together with the coefficient C and the slope γ. For some metals, the simple relation β = 0.55γ yields a good conformity to experimental results [34].

**Figure 9:**
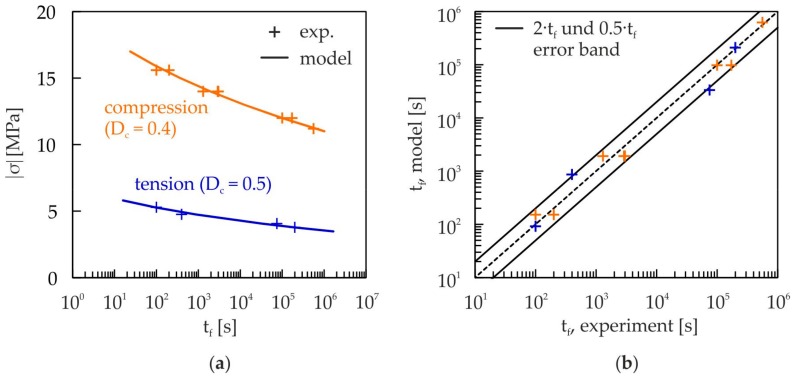
(**a**) Comparison of creep strength lines for static loading (damage model, Equation (5) and experiment); (**b**) Comparison of numerically (damage model, Equation (5)) and experimentally determined lifetimes at the marked load levels in (**a**).

The determination of the stress exponent m for static damage is done in analogy to the procedure in [34]. In the case of compressive damage, the inverse slope of the experimentally determined creep strength line for static loading (see Figure 9a) is used as starting value for m. In the case of tensile damage, it was not possible to achieve fracture, and therefore the creep strength line was determined using D = 0.5 as the failure criterion. In a subsequent parameter variation together with the material parameter A, the best possible conformity with the measured damage evolution and the creep strength lines is searched for.

#### 3.1.3. Material Parameter, A

The material parameter A determines the horizontal position of the creep strength line within the creep diagram. The identification of the material parameter follows after the fixation of the starting value for the stress exponent m. This is an iterative process, in which several pairs of values for the material parameter A and the stress exponent m may deliver a good conformity of the model to the experimental results.

#### 3.1.4. Static Damage Model Parameters

All identified parameters for the static damage model are summarized in Table 2.

#### 3.1.5. Validation of the Static Damage Model

Figure 9 shows the results of the static damage model (Equation (5)) and the experimentally determined lifetimes for tension and compression at different load levels. The calculated lifetimes coincide in nearly all cases, with a deviation below factor 2, with the experimental lifetimes (see Figure 9b). Thus, the damage model is able to predict the lifetime with high accuracy.

### 3.2. Cyclic Damage Model

The cyclic fatigue damage model by Chaboche and Lesne [34] is used for the description of the cyclic damage. In this model, the damage increment dD is calculated as a function of the current damage D, the stress amplitude σ_a_, the stress exponent β, the damage exponent α, and the parameter M for the mean stress dependency, by

(11) $$\mathrm{dD}=D^{\alpha }{\left(\frac{\sigma_{a}}{M}\right)}^{\beta }\mathrm{dN}.$$

Since the damage increment dD depends on the current damage D, the model is able to describe nonlinear damage evolution. Furthermore, it is possible to consider nonlinear damage accumulation (sequence effects) by using the damage exponent α, which depends on the load level.

An integration of Equation (11) for constant stress amplitude and mean stress together with the initial condition D_0_ at N = 0 yields the damage D as a function of the number of cycles

(12) $$D={\left[D_{0}^{1-\alpha }+\left(1-\alpha \right)\;{\left(\frac{\sigma_{a}}{M}\right)}^{\beta }N\right]}^{\frac{1}{1-\alpha }}.$$

Using the critical damage D_c_ at N = N_f_, the fatigue life N_f_ can be calculated as

(13) $$N_{f}=\frac{D_{c}^{1-\alpha }-D_{0}^{1-\alpha }}{1-\alpha }{\left[\frac{\sigma_{a}}{M}\right]}^{-\beta }$$

and the current damage D may be expressed then as a function of the relative fatigue life N/N_f_ as

(14) $$D={\left[D_{0}^{1-\alpha }+\frac{N}{N_{f}}\left(D_{c}^{1-\alpha }-D_{0}^{1-\alpha }\right)\right]}^{\frac{1}{1-\alpha }}.$$

In Equation (14), the damage D depends only on the initial damage D_0_, the critical damage D_c_, and the damage exponent α.

In order to identify the parameters of the cyclic damage model, experiments must be performed in which pure cyclic damage is developed [33]. In general, there is a combination of static and cyclic damage when cyclic experiments are performed. There are two main test parameters by which the damage evolution is influenced: the test frequency and the waveform. Among conventional waveforms (sinus, rectangular, and triangular wave), the triangular wave generates the lowest static damage in the specimen. The static damage portion can also be minimized when the test frequency is sufficiently high. However, the test frequency was limited to 30 Hz in the experiments because of the specimens heating due to hysteretic losses of the material. In case of tensile loading, nearly pure cyclic damage was achieved at 20 Hz test frequency and sinusoidal waveform. In the compressive tests, the test frequency was raised to 30 Hz and a triangular waveform was used [13].

#### 3.2.1. Damage Exponent, α

A nonlinear behavior of the damage evolution curves is taken into account by using the damage exponent α. Since D_0_ and D_c_ are known from experiments (see Figure 7), α remains the only unknown parameter. It can be identified directly by a parameter variation when modeling damage evolution curves with the help of Equation (14) and a comparison with damage evolution curves obtained by experiments. Since α has to be a function of applied stress level because of sequence effects, an appropriate relationship has to be found. It was found in the experiments that there is a good correlation between the damage rate and the maximum stress magnitude |σ| applied in the cyclic tests [13]. The parameter identification is performed using damage evolution curves from compressive loading at R = 10 and from tensile loading at R = 0.1.

**Figure 10:**
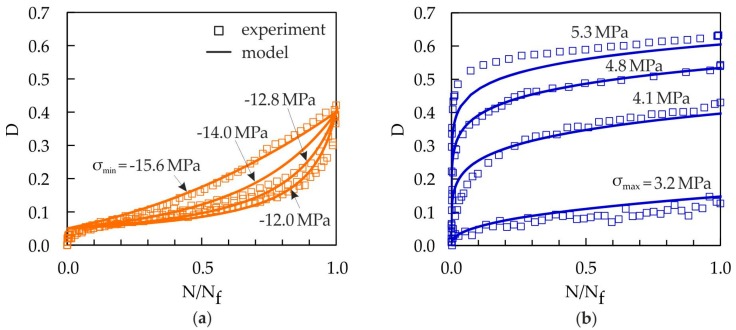
Comparison of damage evolution curves for tensile and compressive loading and pure cyclic damage (experiment and damage model): (**a**) compressive loading (R = 10); (**b**) tensile loading (R = 0.1).

Figure 10 shows the damage evolution curves from compressive and tensile loading experiments in comparison to the damage model represented by Equation (14). An initial damage D_0_ = 0.05 and a critical damage D_c_ = 0.4 are used for all load levels in the compressive loading tests, which results in a good correlation between the experiments and the damage model. For tensile loading, the initial damage D_0_ and the critical damage D_c_ depend on the load level (see Table 3). Also, in the case of cyclic tensile loading, a good correlation between the experiments and the damage model is achieved (see Figure 10).

#### 3.2.2. Stress Exponent, β

The identification of the stress exponent β is performed in analogy to the static stress exponent m (see Section 3.1.2) together with the mean stress parameter M on the basis of experimentally determined Wöhler curves. A variation of the parameters β and M is performed until a good correlation of Wöhler curves and damage evolution curves is achieved.

#### 3.2.3. Mean Stress Parameter, M

In the original version of the cyclic damage model by Chaboche and Lesne, a linear relationship (Goodman relation) between the mean stress and the fatigue limit is used [34]. As shown in [15], this approach cannot be used in case of cellular composite, since the fatigue limit is a highly nonlinear function of the mean stress. Instead, the fatigue limit is approximated using the function

(15) $${M=M}_{0}\;\sigma_{A},$$

where M_0_ is a material parameter, and σ_A_ is the fatigue limit, which is determined experimentally. σ_A_ is approximated using the Gaussian distribution

(16) $$\sigma_{A}=\frac{55\;{\left(\mathrm{MPa}\right)}^{2}}{\sqrt{2\pi }\;3.6\;\mathrm{MPa}}e^{-\frac{1}{2}\frac{\left(\sigma_{m}-\left(-4.6\;\mathrm{MPa}\right)\right){\;}^{2}}{{\left(3.6\;\mathrm{MPa}\right)}^{\;2}}},$$

which leads to a very good correlation with experimental results [13] (see Section 4.1, Figure 12, σ_a_(σ_m_) for N_f_ = 5 × 10^6^). The Gaussian distribution is commonly used for the description of the fatigue limit in the case of fiber reinforced plastics; see e.g., [35]. The material parameter M_0_ is identified together with the stress exponent β with the help of a parameter variation.

#### 3.2.4. Cyclic Damage Model Parameters

The parameters of the cyclic damage model are summarized in Table 3.

#### 3.2.5. Validation of the Cyclic Damage Model

**Figure 11:**
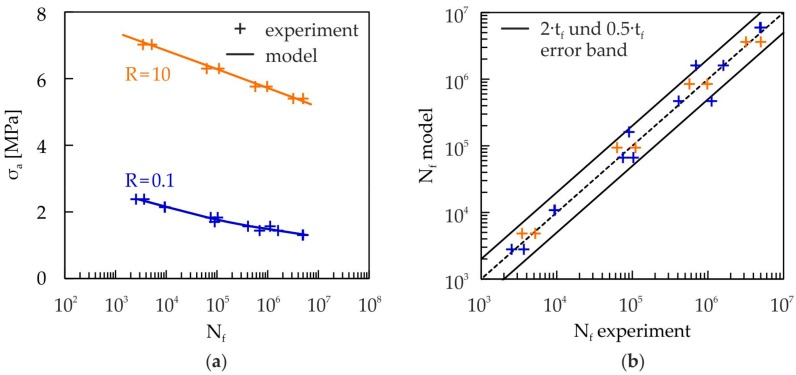
(**a**) Comparison of fatigue lives for cyclic tensile (R = 0.1, f = 20 Hz, sinusoidal waveform) and compressive (R = 10, f = 30 Hz, triangular waveform) loading (experiment and damage model, Equation (13)); (**b**) Comparison of numerically (damage model, Equation (13)) and experimentally determined fatigue lives at the marked load levels in (**a**).

Figure 11 shows the fatigue lives for compressive (R = 10) and tensile (R = 0.1) cyclic loading from the experiments and the damage model for different stress levels. As can be clearly seen from Figure 11a, the fatigue strength of the cellular composite is much higher for compressive than for tensile loading. A very good correlation, within a factor of 2 (see Figure 11b), can be observed for both kinds of loading.

### 3.3. Damage Interaction Model

In case of the cellular composite, static and cyclic damage have the same effect on the microcrack growth in the glass foam granules [9,10,13]. Furthermore, both damage types can interact and therefore accelerate the damage process, which has been observed in the case of many materials [36]. In order to combine the damage portions of static and cyclic damage, the model by Lemaitre and Chaboche [33] is used, in which the damage portions are superposed linearly:

(17) $$dD=dD_{static}+dD_{cyclic}.$$

Since the same damage parameter D is used in both models, it is possible to consider nonlinear damage interaction between the static and the cyclic damage [33].

### 3.4. Numerical Integration

In special cases, a solution of Equation (17) can be obtained analytically [30]. In general, however, the integration of Equation (17) must be carried out numerically. For this purpose, an explicit Euler code has been developed and implemented in Matlab. In the case of pure static damage at constant stress as well as in the case of pure cyclic damage, the step size may be comparatively large, and then the computation times are short. In the case of damage interaction, however, each cycle must be discretized into several smaller steps, since the stress and consequently the static damage rate varies during the cycle. In order to minimize the computation time, a cycle-jumping algorithm has been developed.

At first, Equation (10) is transformed into the time domain by dN → f × dt. After that, the initial value problem (Equation (17))

(18) $$\dot{D}=f(t,D),\;D(t=0)=D_{0}$$

is integrated in the time domain by using the time step size Δt by

(19) $$D(t+\Delta t)=D(t)+\;\dot{D}(t)\;\Delta t.$$

A discretization of a single cycle into 100 equidistant time steps has proven to be sufficient for high accuracy. After this step, the damage increment per cycle ΔD/ΔN is obtained.

In the next step, it is checked if the damage increment is higher or lower than a predefined threshold level:

(20) $$\frac{\Delta D}{\Delta N}\leq \frac{D_{c}}{500}.$$

If the damage growth is lower than or equal to D_c_/500, the cycle jumping will be activated. This threshold value represents a good agreement between accuracy and computation costs. The number of omitted cycles is computed by

(21) $${\Delta N}_{\mathrm{jump}}=\frac{D_{c}}{\Delta D/\Delta N\;\;500}$$

and rounded to integer values. Finally, the damaged D is updated by

(22) $$D(N+{\Delta N}_{\mathrm{jump}})=D(N)+\frac{\Delta D}{\Delta N}\;{\Delta N}_{\mathrm{jump}}.$$

The lifetime t and the number of cycles N will also be updated by t + Δt_jump_ and N + ΔN_jump_. A linear damage growth is supposed during cycle-jumping. In the case when the damage rate is high and the damage growth in a single loading cycle is higher than the threshold value, the damage computation is continued by the next cycle.

This procedure is repeated until the critical damage is reached. It allows for a very fast calculation of the damage evolution for constant load frequencies. The computation time is reduced by a factor of 10 to 10,000, depending on the number of cycles to failure [13].

## 4. Damage Model Application

### 4.1. Mean Stress Effect in Pure Cyclic Damage

Structures or structural parts have to endure fluctuating stresses, which are characterized by their amplitude and mean components. When applying lifetime prediction for cyclic loading, the influence of the mean stress on the endurable amplitude for a particular lifetime is important. A convenient graphical representation of different combinations of amplitude and mean stresses in relation to various fatigue lifetimes is the constant-life fatigue diagram. In this diagram, lines of constant fatigue lives are plotted. The creation of such a diagram on a pure experimental basis requires a lot of time-consuming and expensive material tests. It is advantageous to create a constant-life fatigue diagram on the basis of a limited amount of material tests with satisfactory accuracy in an efficient way. This can be done with the help of the proposed damage model.

**Figure 12:**
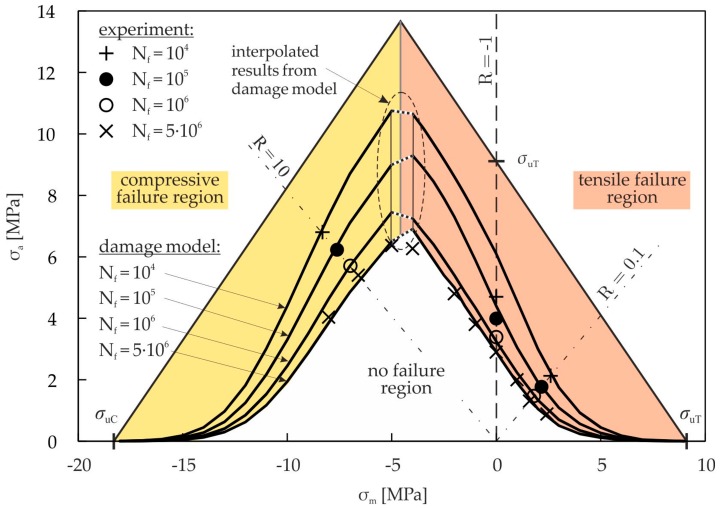
Constant-life fatigue diagram; damage model (Equation (13)) and experimental values.

Figure 12 shows the constant-life fatigue diagram for the cellular composite. The lines of constant fatigue lives are calculated using the continuum damage model for pure cyclic damage via Equation (13). The stress amplitudes in the region of σ_m_ ≤ −5 MPa are calculated with the model parameters for compressive damage, and those in the region of σ_m_ ≥ −4 MPa are calculated with the model parameters for tensile damage. The values of the region −5 MPa < σ_m_ < −4 MPa are linearly interpolated (see Figure 12). This avoids the discontinuity which would otherwise occur. In addition, the experimentally determined results for constant R-values are drawn in Figure 12.

The damage model results in approximately symmetric finite and infinite life fatigue strength curves. The infinite life fatigue strength at N_f_ = 5 × 10^6^ cycles is represented by the damage model with high precision except for a small range at about σ_m_ = −4 MPa. In this range, the model for tensile damage results in slightly higher endurable amplitudes compared to the experimental values. The finite fatigue life strength values for R = 10 and R = 0.1 coincide with the experimental data, since the parametrization of the damage model is based on these R-values. For reversed loading (R = −1), the endurable amplitudes at high lifetimes are approximated by the model very accurately. At lower lifetimes (N_f_ ≤ 10^5^), the model provides higher endurable amplitudes in comparison to the experimental results. The reason is the fact that the damage model was developed and validated for cyclic tensile (R = 0.1) and compressive (R = 10) loading. To increase the accuracy of the model, more R-values could be taken into account for the parameterization of the damage model. Within the model, either tensile or compression damage is considered. Especially in the range of about σ_m_ = −4.5 MPa, both tensile and compressive damage may appear as shown in [13]. Thus, a higher total damage within the cellular composite occurs, which is not included in the model. Further research work is needed to adequately describe the damage evolution in this region.

### 4.2. Frequency Effect in Static and Cyclic Damage Interaction

**Figure 13:**
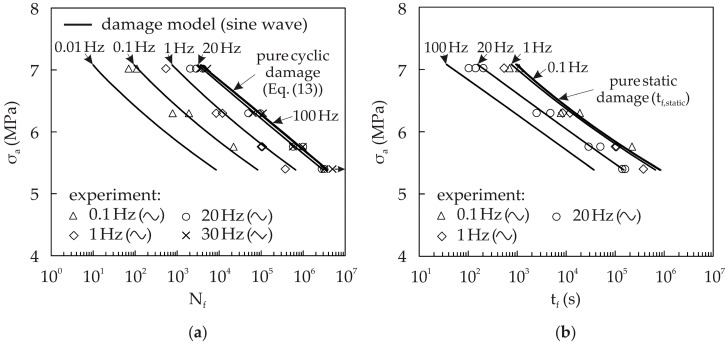
Effect of loading frequency in the case of compressive cyclic loading (R = 10) in terms of (**a**) cycles to failure N_f_; and (**b**) time to failure t_f_.

During a cyclic loading on a cellular composite, there are static and cyclic damage processes in progress. The superimposed static damage results in a reduced lifetime in comparison with the pure cyclic damage. The influence of the static damage depends on the test frequency and the waveform. To demonstrate the capability of the damage model, the lifetimes for compressive cyclic loading (R = 10) at different frequencies and waveforms are calculated and compared with experimental results. Experimental results are available for the sinusoidal waveform with 0.1 Hz, 1 Hz, and 20 Hz, and for the triangular waveform with a 30 Hz test frequency. Figure 13 shows that there is a strong influence of frequency on lifetime in terms of the number of cycles to failure N_f_ and the time to failure t_f_, which is obtained by dividing the number of cycles N_f_ by the test frequency f. The results from the damage model agree well with the experimental results.

The experimental Wöhler curve for nearly pure cyclic damage was created using a triangular waveform with f = 30 Hz, and is the basis for the identification of the parameters for the cyclic damage model. Therefore, the calculated cycle numbers (Equation (13)) represent an upper limit for the achievable cycles to failure. The cycle numbers in Figure 13a for superimposed static and cyclic damage are consequently always below these cycle numbers. Using a higher frequency, e.g., f = 100 Hz, there is no further increase of the cycle numbers. When reducing the test frequency, the amount of static damage increases and the cycles to failure N_f_ decrease accordingly. It can be seen in Figure 13a that the cycles to failure N_f_ decrease continuously by reducing the test frequency, because the static damage gains more influence. In contrast, the fatigue life for f = 100 Hz is only slightly higher than that for f = 20 Hz. This means that the influence of static damage becomes lower at higher frequencies.

With the help of the damage model (Equation (17)), the time to failure t_f,static_ for the amount of pure static damage at cyclic loading can be calculated, when setting the term for the cyclic damage to zero. This results in a time to failure t_f,static_ which is for the same waveform independent of the frequency, and represents an upper limit for the accessible time to failure t_f_. Figure 13b shows that almost pure static damage is present for f = 0.1 Hz. The corresponding Wöhler line coincides with the Wöhler line for pure static damage, which is represented by the time to failure t_f,static_. A further reduction of the frequency does not lead to an increasing time to fracture t_f_. Therefore, the frequency f = 0.1 Hz represents the lower frequency limit, where static damage is dominant. The time to failure at higher frequencies is shorter than that of lower frequencies, because more cyclic damage is accumulated.

**Figure 14:**
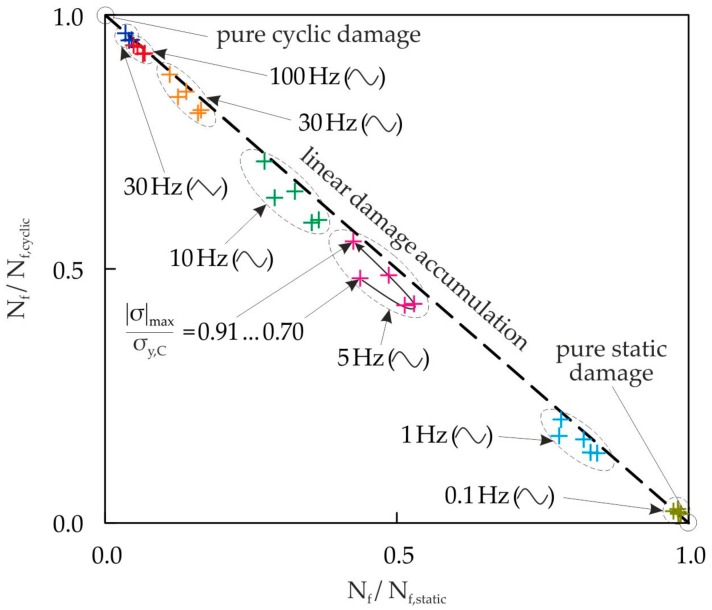
Calculated portions of static and cyclic damage with different waveforms and frequencies in the case of compressive cyclic loading (R = 10) for different load levels.

The influence of the static and cyclic damage interaction on the cycles to failure is illustrated in Figure 14. The position of the frequency limits, where only either static or cyclic damage is present, can be analyzed by an examination of the static and cyclic damage portions for the corresponding test signal and frequencies. For this reason, the cycles to fracture N_f_ for different frequencies and waveforms are calculated via Equation (17) and normalized by the cycles to fracture for pure static damage N_f,static_ or the cycles to fracture for pure cyclic damage N_f,cyclic_, and are drawn as an interaction diagram in Figure 14. This diagram illustrates the portions of static damage or cyclic damage of the total damage. The cycles to fracture for pure cyclic damage N_f,cyclic_ are calculated with Equation (13). The cycles to fracture for pure static damage N_f,static_ are determined using t_f,static_ and the frequency f, with

N_f,static_ = t_f,static_ f. (23)

The lifetimes in Figure 14 are evaluated for the load range of |σ|_max_/σ_y,C_ = 0.70…0.91 in five equidistant steps for all selected waveforms and frequencies. The interaction diagram shows that the cyclic damage is dominant for the triangular waveform with f = 30 Hz. The amount of static damage is, on average, below 3%. Therefore, there is nearly pure cyclic damage present. In the case of the sinusoidal signal with f = 0.1 Hz, static damage dominates.

The relation between the pure static and pure cyclic damage varies for a constant frequency and waveform with the load level. At the frequency f = 5 Hz, the largest variation occurs. For higher or lower frequencies, the variation gets smaller and vanishes at pure static and pure cyclic damage. Figure 14 shows further that an approximately linear accumulation or interaction of the static and cyclic damage occurs. Some materials, such as the austenitic steels or the nickel base alloys show strong nonlinear damage accumulation under creep-fatigue loading conditions, which reduces their lifetime by a factor of 2 to 10 compared with the lifetime prediction using linear damage accumulation [37]. For the presented cellular composite, a nearly linear damage accumulation or interaction is present, which is positive for the lifetime when the static and cyclic damage is superimposed.

### 4.3. Influence of the Waveform

**Figure 15:**
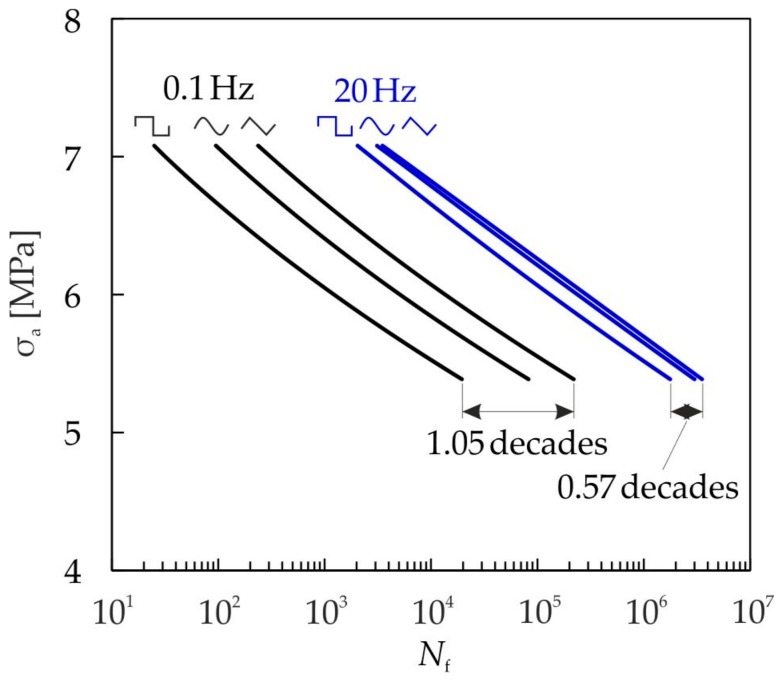
Influence of the waveform and frequency on the cycles to fracture N_f_ for compressive cyclic loading (R = 10).

For the pure cyclic damage, the lifetime is not dependent on the waveform since only the stress amplitude, the mean stress, and the number of cycles contribute to damage accumulation. In the case of superimposed static and cyclic damage, the waveform has a significant influence on the attainable cycles to failure. In the following, the damage model (Equation (17)) is used to evaluate the influence of the waveform on the lifetime calculation. The calculated Wöhler lines for cyclic compression loading (R = 10) with different waveforms and the frequencies f = 0.1 Hz and f = 20 Hz are plotted in Figure 15.

Using the triangular waveform, the highest cycle numbers are obtained at both frequencies. The rectangular waveform yields the lowest lifetime, because here the accumulated time at a high stress level, and consequently the static damage, is the highest. The influence of the waveform is stronger for f = 0.1 Hz compared with f = 20 Hz. With an increasing frequency over 20 Hz, the influence of the waveform vanishes (see Figure 13a). Further results, including an investigation of different load ratios and sequence effects, are discussed in [13].

## 5. Conclusions

The fatigue behavior of a cellular composite was analyzed by a microstructural investigation using scanning electron microscopy and the mechanical properties for static and cyclic uniaxial loading are presented. A continuum damage mechanics model for cellular composites under static and cyclic loading was developed. The damage is modeled as an interaction between static and cyclic damage, and the model incorporates the nonlinear accumulation and interaction of damage. A cycle-jumping procedure is presented, which allows very fast computation times of the damage evolution and lifetimes.

Using the cyclic damage model, it is possible to consider the mean stress effect. To demonstrate the model capabilities, a constant-life fatigue diagram was calculated and compared with experimental results. A very good correlation could be achieved for the infinite life fatigue strength and for finite life fatigue strength at R = 10 and R = 0.1 stress ratios. Further work is needed to adequately model the damage behavior at mean stresses of about −4.5 MPa, where both compressive and tensile damage contribute to the damage process at the same time. In general, more R-values should be investigated in future works in order to increase the accuracy of the damage model.

The lifetimes for different frequencies and compressive loadings were calculated by means of the damage interaction model. It was shown that the number of cycles to failure decreases with lower frequencies. There is a lower frequency limit, below which the time to fracture t_f_ does not decrease anymore. On the other hand, there is an upper frequency limit, above which the number of cycles to fracture N_f_ does not depend on the frequency. Between the lower and the upper frequency limit, there is always an interaction between the static and the cyclic damage. In addition, it was shown that the damage interaction between the static and cyclic damage is less pronounced, and that there is almost a linear damage accumulation present.

As a last example, the influence of the waveform was investigated using the damage interaction model. It was shown that the influence of the waveform depends on the loading frequency, and is more pronounced at lower frequencies. A good agreement with experimental results was achieved.

## Figures and Tables

**Table 1:** Mechanical properties of the cellular composite with 1–2 mm glass foam granules (data from [13]).

Density ρ (g/cm^3^)	Young’s Modulus E (MPa)	Poisson’s Ratio ν (–)	Tensile Yield Strength σ_y,T_ (MPa)	Tensile Strength σ_u,T_ (MPa)	Compressive Yield Strength σ_y,C_ (MPa)	Compressive Strength σ_u,C_ (MPa)
0.72	3098	0.31	5.8	9.1	17.1	18.3

**Table 2:** Parameters for static damage model.

Loading	A	m	p	D_0_	D_c_
compression	4.5 × 10^−39^	30	−1.8 |σ|/(MPa) + 27.5	0.05	0.4
tension	7.0 × 10^−29^	40	−4.5 σ/(MPa) + 8.0	Equation (2)	0.5

**Table 3:** Parameters for cyclic damage model.

Damage in	α	β	M_0_	D_0_	D_c_
compressive region	−0.43 |σ|_max_/(MPa) + 7.3	4.75	12.7	0.05	0.4
tensile region	−3.2 σ_max_/(MPa) + 8.8	4.5	90.9	Equation (2)	Equation (3)
